# Cloning, expression and characterization of a pectate lyase from *Paenibacillus* sp. 0602 in recombinant *Escherichia coli*

**DOI:** 10.1186/1472-6750-14-18

**Published:** 2014-03-10

**Authors:** Xiaoman Li, Huilin Wang, Cheng Zhou, Yanhe Ma, Jian Li, Jiangning Song

**Affiliations:** 1 National Engineering Laboratory for Industrial Enzymes and Key Laboratory of Systems Microbial Biotechnology, Tianjin Institute of Industrial Biotechnology, Chinese Academy of Sciences, Tianjin 300308, China; 2 State Key Laboratory of Microbial Resources, Institute of Microbiology, Chinese Academy of Sciences, Beijing 100101, China; 3 Drug Delivery, Disposition and Dynamics, Monash Institute of Pharmaceutical Sciences, Monash University, Parkville Campus, 381 Royal Parade, Parkville, Victoria 3052, Australia; 4 Department of Biochemistry and Molecular Biology and ARC Centre of Excellence in Structural and Functional Microbial Genomics, Faculty of Medicine, Monash University, Clayton, Victoria 3800, Australia

**Keywords:** Pectate lyase, High-level expression, Characterization, Degumming

## Abstract

**Background:**

Biotechnological applications of microbial pectate lyases (Pels) in plant fiber processing are considered as environmentally friendly. As such, they become promising substitutes for conventional chemical degumming process. Since applications of Pels in various fields are widening, it is necessary to explore new pectolytic microorganisms and enzymes for efficient and effective usage. Here, we describe the cloning, expression, characterization and application of the recombinant Pel protein from a pectolytic bacterium of the genus *Paenibacillus* in *Escherichia coli*.

**Results:**

A Pel gene (*pel*N) was cloned using degenerate PCR and inverse PCR from the chromosomal DNA of *Paenibacillus* sp. 0602. The open reading frame of *pel*N encodes a 30 amino acid signal peptide and a 445 amino acid mature protein belonging to the polysaccharide lyase family 1. The maximum Pel activity produced by *E. coli* in shake flasks reached 2,467.4 U mL^−1^, and the purified recombinant enzyme exhibits a specific activity of 2,060 U mg^−1^ on polygalacturonic acid (PGA). The maximum activity was observed in a buffer with 5 mM Ca^2+^ at pH 9.8 and 65°C. PelN displays a half-life of around 9 h and 42 h at 50°C and 45°C, respectively. The biochemical treatment achieved the maximal reduction of percentage weight (30.5%) of the ramie bast fiber.

**Conclusions:**

This work represents the first study that describes the extracellular expression of a Pel gene from *Paenibacillus* species in *E. coli*. The high yield of the extracellular overexpression, relevant thermostability and efficient degumming using combined treatments indicate its strong potential for large-scale industrial production.

## Background

Pectinases have various important applications in plant fiber processing, especially in textile, paper, fruit juice, coffee, and tea fermentation industries [1,2]. Depending on the mode of action and substrate specificity, pectinases involved in such biotechnological interventions can be classified into three major groups: protopectinases, pectinesterase (EC 3.1.1.11), and pectin depolymerases [3]. Among the pectin depolymerases, pectate lyase (Pel, EC 4.2.2.2) has attracted a great deal of interest in the past few decades from both scientific and commercial perspectives. Pel is capable of cleaving α-1,4 linkages in pectate polymers by β-elimination mechanism, producing 4,5-unsaturated oligogalacturonates [4]. Pectin, composed of partially methylesterified pectate, is more commonly used as a natural substrate in fiber crop degumming and retting processes, as it is an essential component in the middle lamella and the primary cell wall of higher plants [5]. Therefore, Pels that are capable of depolymerizing methylesterified substrates are more useful for industrial application. For instance, in textile processing, a crucial step for successful scouring of fabric is wax and pectin removal [6], which is generally performed under high temperature and high alkaline conditions that cause serious environment impacts and raw material loss. Therefore, it is desirable to develop Pels with high thermostability and alkalistability, which can be used as the ideal candidates for both commercially viable and environmentally responsible bioprocesses.

Pectolytic enzymes are ubiquitous in nature, and most of the Pels previously reported have been isolated from microorganisms such as bacteria and fungi [7]. Industrial applications of bacterial alkaline Pels, the majority of which occur in the *Bacillus* and *Pseudomonas* genus, have been extensively studied [1,3]. However, in terms of practical bioprocesses, only a few of them are robust enough to undergo long-time bioprocess treatment. Recently, enzymes originating from thermophilic or alkaliphilic microorganisms are garnering more interest [8-10] because of their good performance at extreme temperature or pH. Although only limited information on the alkaliphilic strains of *Paenibacillus* spp. exists, several Pels from this genus have proved to be highly stable [10-12]. Hence, these bacteria have great potential for industrial-scale production of novel enzymes with desirable compatibility between the optimal temperature/pH and the extended useful life.

In the present study, we cloned the gene of a Pel (PelN) from *Paenibacillus* sp. 0602 isolated and identified from high alkaline soil based on its capacity of producing Pel. The biochemical properties of the recombinant PelN were comprehensively studied, which suggested its tolerance to high temperature, alkaline condition, and methylated substrates. The production of the recombinant PelN was accomplished in an efficient *Escherichia coli* secretory expression system, which achieved the highest yield in shake flasks reported so far with a high secretion capability. The biochemical degumming experiments of PelN suggested its favourable efficiency in ramie fiber bast degumming and various potential applications in biotechnology processes, especially in textile industry.

## Results

### Sequence analysis

A DNA fragment of 128 bp was obtained in degenerate PCR. The subsequent BLAST search in GenBank suggested that this fragment was a part of a Pel gene. The assembly of this fragment with the sequence obtained in inverse PCR with specific primers of this fragment produced a nucleotide of 2740 bp which consisted of the complete ORF of a Pel gene (*pel*N) and its flanking fragments. A putative promoter was predicted to be in the upstream region of the ORF, and the −35 and −10 regions were “5′-TTTACA-3′” and “5′-TATTAT-3′,” respectively. A typical ribosomal binding site (5′-AGGAGG-3′) was also located upstream of the ATG initial codon. In addition, in the downstream of the ORF, a rho-independent transcription terminator was also identified.

**Figure 1:**
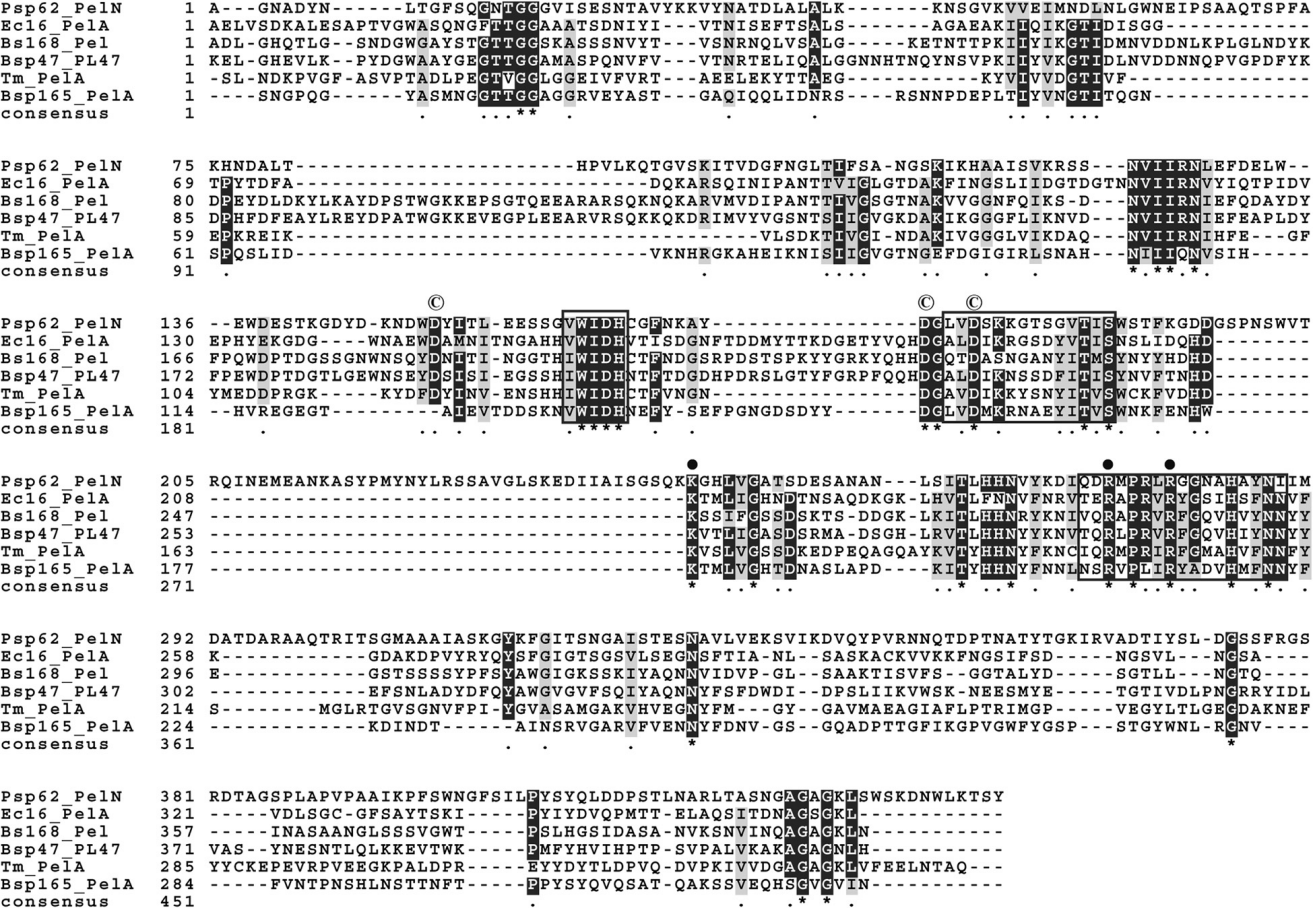
**Multiple sequence alignment of PelN with several Pels of the PL1 family with known structures.** Psp62_PelN (*Paenibacillus* sp. 0602 PelN), Ec16_PelA (*Erwinia chrysanthemi* EC16 PelA), Bs168_Pel (*B. subtilis* subsp. *subtilis* 168 Pel), Bsp47_PL47 (*Bacillus* sp. TS-47 PL47), Tm_PelA (*Thermotoga maritima* PelA), and Bsp165_PelA (*Bacillus* sp. N165 PelA). Numbering begins at the N-terminus of the proteins without signal peptides. Identical and conserved amino acids are highlighted by black and gray, respectively, while gaps are indicated by dashes. Conserved sequence patterns of Pels are denoted by boxes. In the final line, asterisks indicate conserved sites while dots denote semi-conserved sites. Conserved catalytic residues and putative Ca^2+^-binding residues of PelN are denoted by black circle and © symbol, respectively.

The deduced protein sequence of the ORF was found to have 475 amino acids, including a typical signal sequence cleaved by the type I signal peptidase (SPase I) [13]. The signal peptide of 30 amino acids comprised a positively charged N-domain with lysine and arginine residues, a hydrophobic H-domain with 24 amino acids, and a SPase I cleavage site of three amino acids. It was found that the downstream residues of the signal peptide at the N-terminus of the mature protein also contributed to the cleavage site recognition [14]. The consensus sequence G(Aro)a(3-7×)GG of Pels, which was predicted to form the cleavage signal at the mature protein side [15], was also present in the N-terminus of PelN in the form of G(Aro)a(6)GG. Analysis of the MSA of PelN and other Pels with solved structures from the PL1 family identified three conserved sequence patterns and a set of invariant amino acids located within the PelN peptide (Figure 1). The conserved sequence patterns used to characterize a novel sequence as a Pel are AxDIKGxxxxVTxS, vWiDH, and VxxRxPxxRxGxxHxxxN [15]. The invariant amino acids involved in catalytic function mainly comprised the catalytic residues R275, K244, and R280, and the Ca^2+^-binding residues D151, D173, and D177 in PelN. Six single mutants of these key residues, obtained by site-directed mutagenesis, resulted in almost completely inactive proteins, as shown in [Additional file 1].

According to the CAZy database ( http://www.cazy.org/Polysaccharide-Lyases.html, accessed in December 2013), Pels occur within five PL families: PL1, PL2, PL3, PL9, and PL10. The BLAST (blastp) search against the NCBI database indicated that PelN is the closest to the PL1 family sequences. The deduced amino acid sequence of PelN exhibited 67-90% identity with the corresponding amino acid sequences from the same genus (YP_005074750, WP_007428224, WP_010348601, WP_017425629, WP_002622431 and ADB78775).

### Expression and purification of PelN

**Figure 2:**
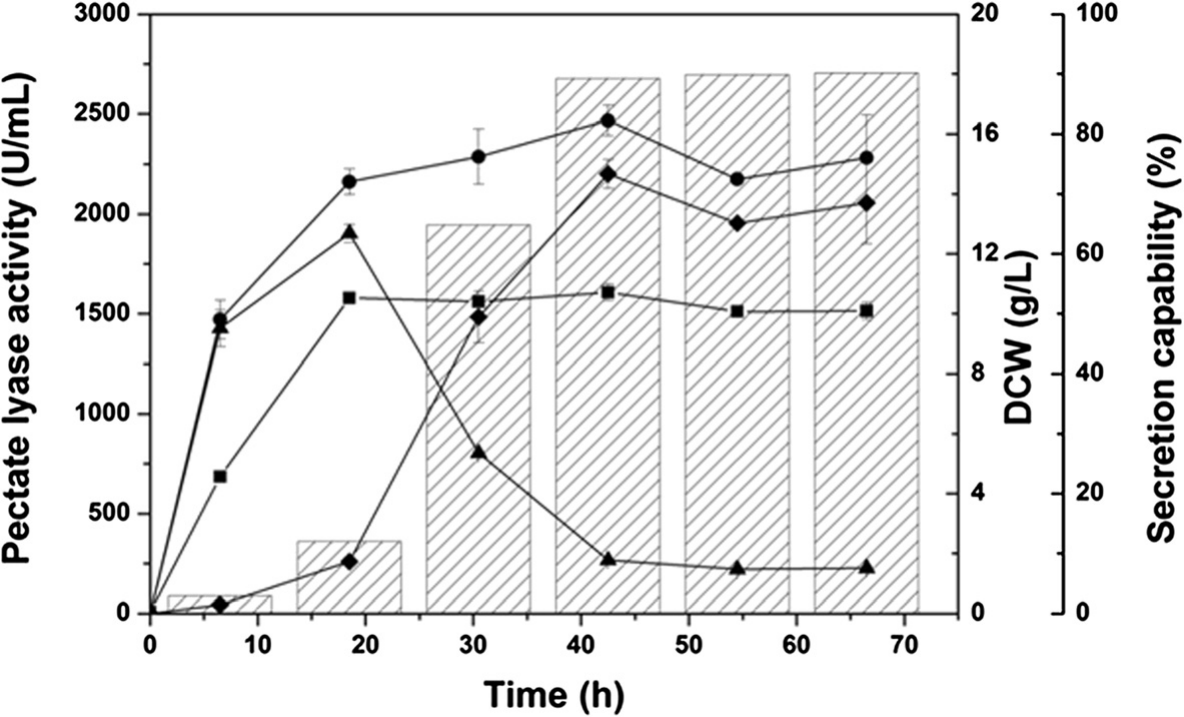
**Time profiles for shake-flask cultivation in 250-mL flasks.** Intracellular Pel activity (▲), extracellular Pel activity (♦), total Pel activity (●), cell concentration (■), and secretion capability (sparse column). Error bars represent ± SD (n = 3).

PelN without the signal sequence was successfully expressed in the recombinant *E. coli* BL21 (DE3) in shake-flask cultivation mode (Figure 2). Under the optimal induction condition in this case (50 mM isopropylthio-β-galactoside, 25°C), the highest extracellular and total Pel activities reached 2,201.7 and 2,467.4 U mL^−1^ after 42-h cultivation, respectively, with a high secretion capability of 89.2%.

**Figure 3:**
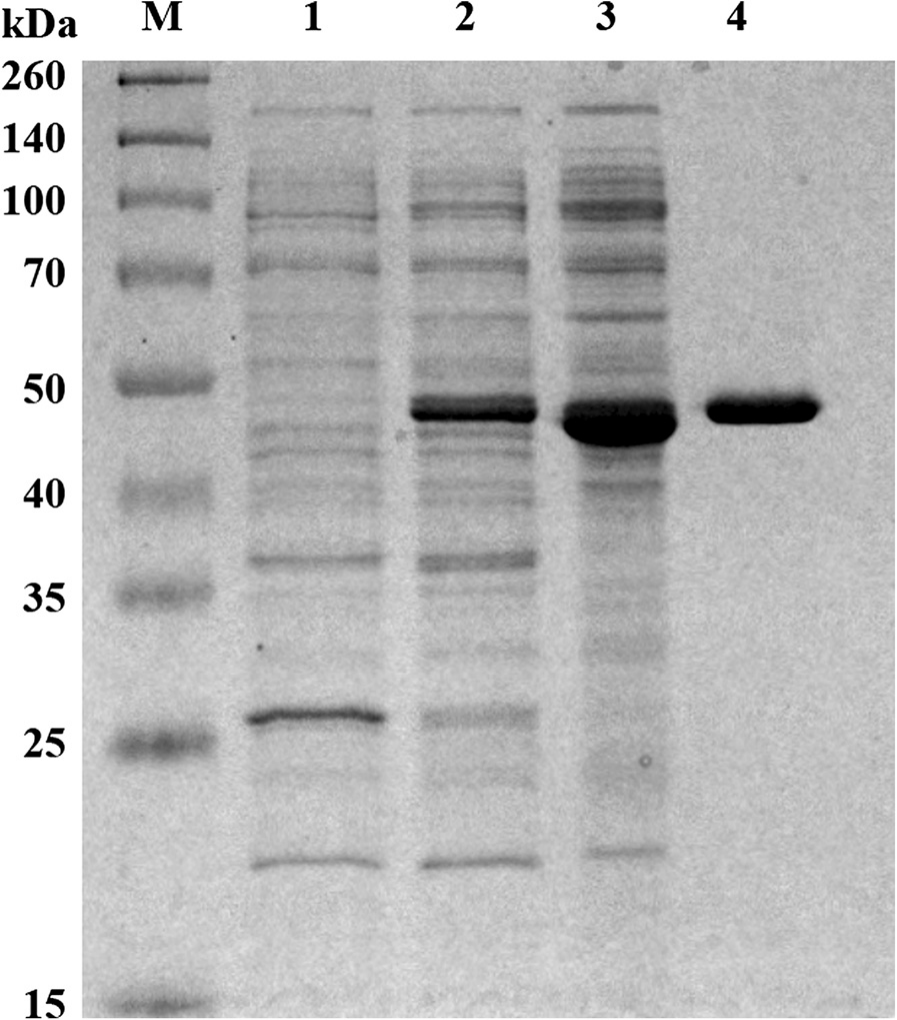
**The recombinant PelN on an SDS-PAGE gel.** Lanes: M, molecular standard; 1, supernatant of the crude extract from *E. coli* BL21 (DE3) harbouring pET22b; 2, supernatant of the crude extract from *E. coli* BL21 (DE3) harbouring pET-pelN; 3, after ammonium sulphate precipitation and resuspension; and 4, purified recombinant PelN.

PelN was purified from the crude extract of *E. coli* BL21 (DE3) harbouring pET-pelN. The two-step purification resulted in a three-fold increase in purity with a yield of 29%, and the purified protein had a specific activity of 2,060 U mg^−1^ on PGA (Table 1). Furthermore, PelN generated a single electrophoretic band on the SDS-PAGE gel at 48 kDa, corresponding to the calculated molecular weight (Figure 3).

**Table 1:** **Purification of the recombinant PelN from *E. coli *** BL21 (DE3) *

**Fraction**	Activity ** (kU)	**Protein (mg)**	**Specific activity (U mg**^**−1**^**)**	**Purification (fold)**	**Yield (%)**
Crude extract of supernatant	74.2 ± 0.6	109.8 ± 0.9	675.8 ± 55.9	1	100
Ammonium sulphate precipitation	46.6 ± 6.2	39.9 ± 3.9	1,166.5 ± 59.8	1.73	62.75
UNOsphere S column	21.5 ± 0.1	10.5 ± 0.3	2,060.0 ± 71.1	3.05	29.02

*Data represent means ± SD (n = 4) relative to the reference samples;

**The activity was measured on PGA under the standard conditions.

### Recombinant enzyme characterization

#### Optimum pH and temperature

**Figure 4:**
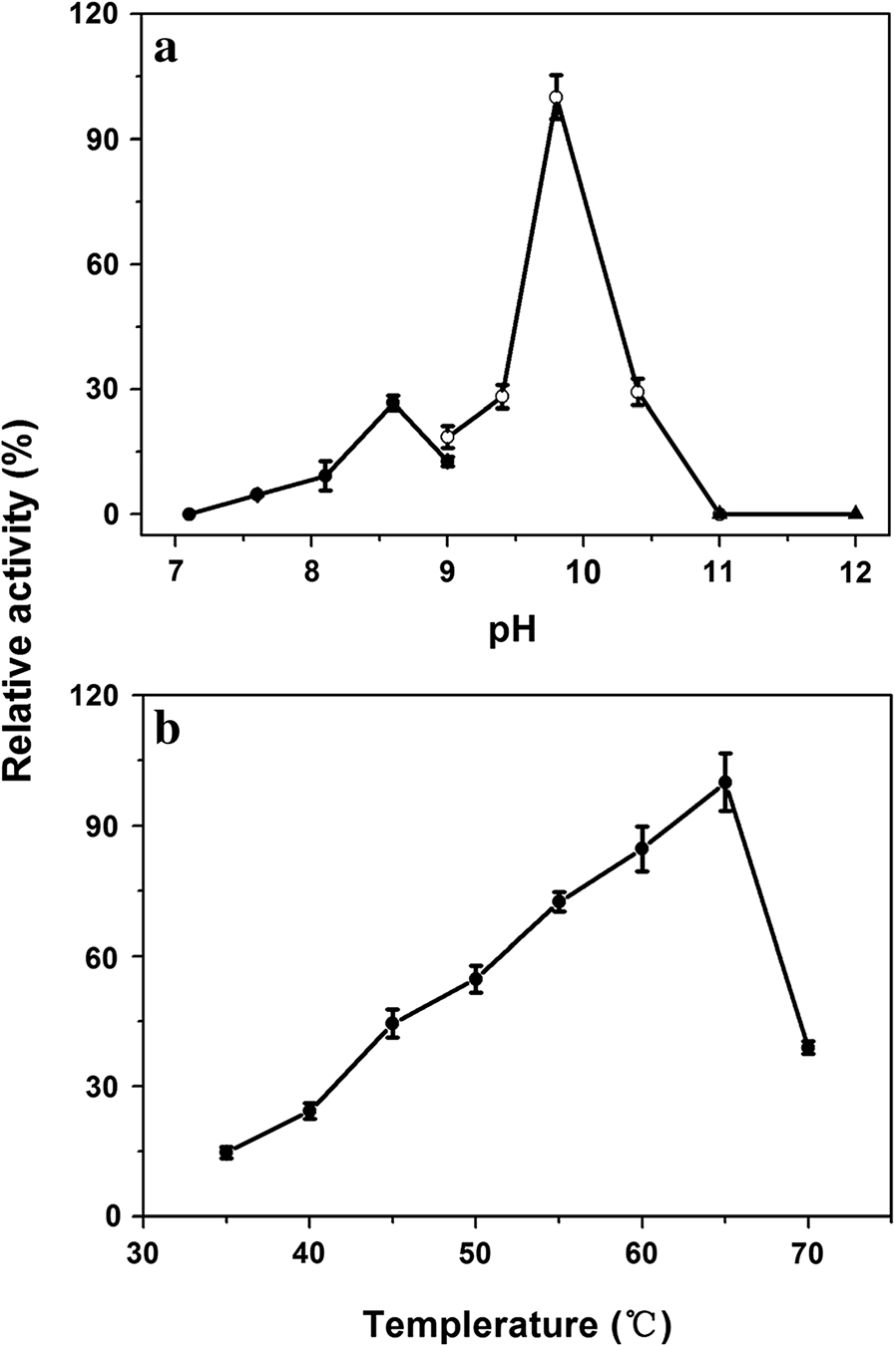
**Effects of pH and temperature on the activity of the purified recombinant PelN. (A)** The optimal pH was determined at 45°C in 50 mM Tris–HCl buffer (pH 7.1–9.0, ■), 50 mM glycine–NaOH buffer (pH 9.0–11.0, ○), and 50 mM Na_2_HPO_4_–NaOH buffer (pH 11.0–12.0, ▲); **(B)** the optimal temperature was determined at pH 9.8. The highest specific activities in both the assays were respectively used as references (100%). Error bars represent ± SD (n = 4).

The purified PelN displayed activity over a wide range of pH from 7.6 to 10.4, with a maximum activity at pH 9.8 (Figure 4a). The effect of temperature on the PelN activity was measured at the optimal pH of 9.8 and different temperatures ranging from 35 to 70°C. The Pel activity increased gradually from 14.8% (682.8 U/mg) at 35°C to the maximum of 100% (4629.2 U/mg) at 65°C and then decreased sharply to 38.9% (1801.7 U/mg) at 70°C and 18.0% (831.9 U/mg) at 75°C (Figure 4b).

#### The effects of different metal ions and chemicals

**Figure 5:**
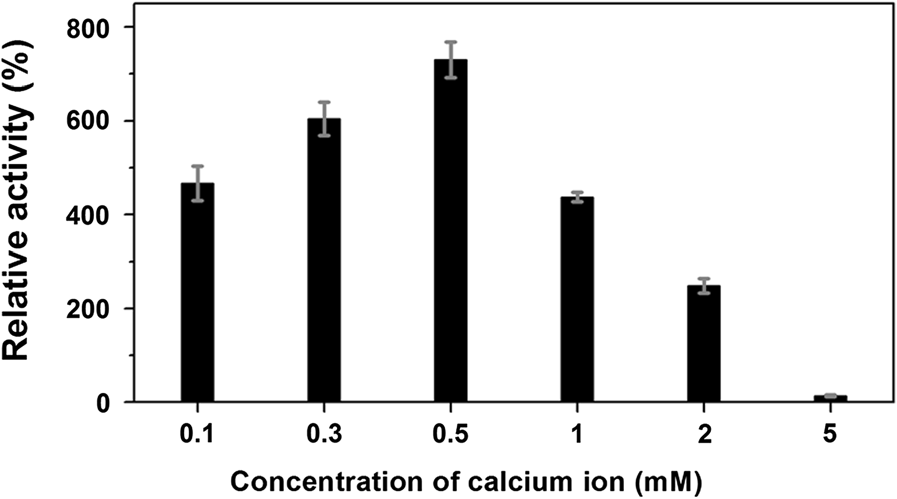
**Effects of different Ca**^**2+ **^**concentrations on the activity of the purified recombinant PelN.** The assays were performed under pH 9.8 at 45°C with the addition of different concentrations of Ca^2+^. The activity under the standard conditions without any chemicals was used as a reference (100%). Error bars represent ± SD (n = 4).

The effects of various metal ions and chemicals were determined under the standard reaction condition. We found that PelN exhibited robust tolerance to several metal ions, and only Ba^2+^ caused a severe loss of activity (Table 2). On the other hand, Triton X-100 and Tween-20 had negligible influence on the activity (Table 2). Similar to the majority of Pels, PelN required Ca^2+^ for its maximal activity. The presence of 0.5 mM Ca^2+^ in PL buffer (50 mM glycine–NaOH buffer, pH 9.8) increased enzyme activity by more than 7-fold, while no activity was detected with the addition of 0.5 and 1 mM EDTA, indicating that Ca^2+^ plays an important role in the catalytic reaction of PelN. Hence, we also determined the optimal Ca^2+^ concentration (Figure 5). In particular, PelN activity was found to be considerably elevated with the addition of 0.1–2 mM Ca^2+^, but was inhibited at a high concentration of more than 5 mM Ca^2+^.

**Table 2:** Effects of different chemicals on the activity of the purified recombinant PelN

**Chemicals**	Relative activity (%)*	
Concentration (mM)	**0.5**	**1.0**
None	100 ± 1	100 ± 1
Ca^2+^	729 ± 38	437 ± 1
Mg^2+^	125 ± 11	99 ± 3
Mn^2+^	75 ± 3	57 ± 5
Cu^2+^	79 ± 3	104 ± 11
Zn^2+^	116 ± 8	106 ± 7
Fe^2+^	82 ± 7	57 ± 3
Ba^2+^	12 ± 3	3 ± 0
K^+^	119 ± 8	100 ± 4
EDTA	nd	nd
Triton X-100	102 ± 9	100 ± 8
Tween-20	98 ± 2	116 ± 11

nd: no detectable activity.

*The activity under the standard conditions without any chemicals was used as the reference (100%), and data represent means ± SD (n = 4) relative to the untreated reference samples;

#### Protein stability

**Figure 6:**
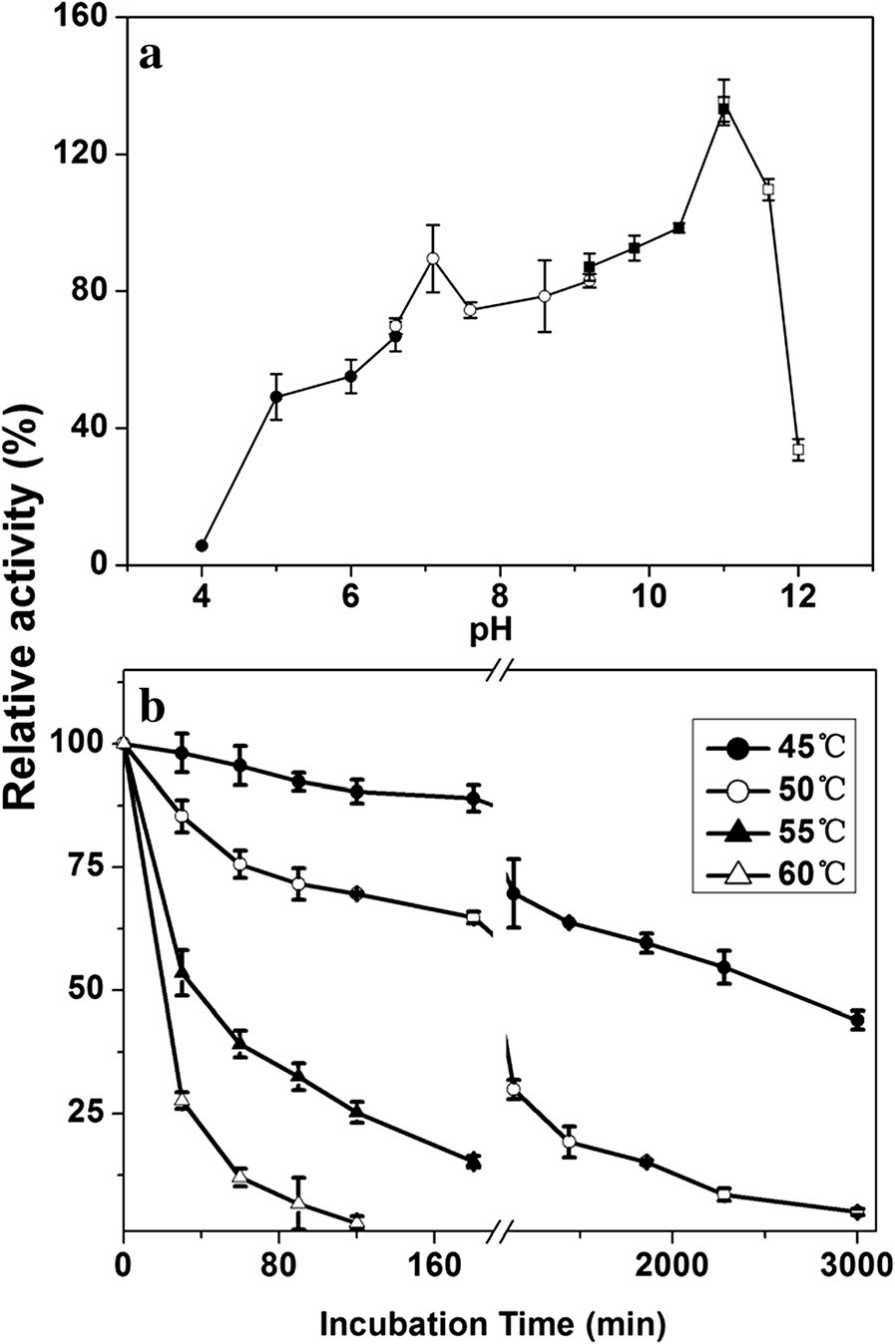
**The alkalistability (a) and thermostability (b) of PelN. (a)** The pH-dependent stability was determined by incubation at 45°C for 60 min in 50 mM sodium citrate–citric acid buffer (pH 4.0–6.6, ●), 50 mM Tris–HCl buffer (pH 6.6–9.2, ○), 50 mM glycine–NaOH buffer (pH 9.2–11.0, ■), and 50 mM Na_2_HPO_4_–NaOH buffer (pH 11.0–12.0, □); **(b)** Thermostability was determined by incubation at 45°C (●), 50°C (○), 55°C (▲), and 60°C (△), respectively. The measured specific activity of PelN without treatment was used as reference (100%), and all reactions were performed under the standard conditions. Error bars represent ± SD (n = 4).

As the fiber degumming process could be accelerated under alkaline conditions [2], a favourable alkalistability of Pels is essentially required for many industrial processes. To explore this potential, the pH-dependent stability of the enzyme was further investigated by pre-incubation for 1 h at 45°C at varying pH values (Figure 6a). The purified PelN was found to be stable over a pH range of 7.1–11.6 and was most stable at pH 11.0.

**Figure 7:**
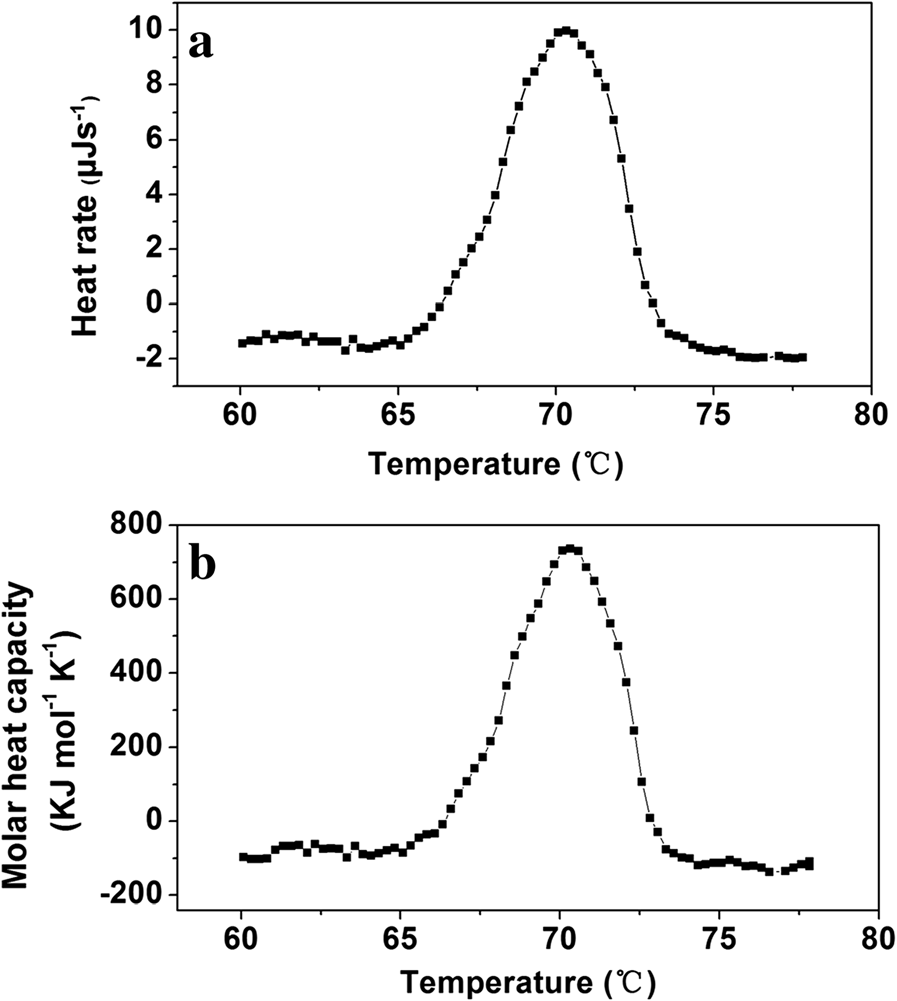
**DSC of the purified PelN. (a)** Raw DSC data curve after correcting the buffer–buffer scanning baseline; **(b)** Conversion of the raw DSC signal to the molar heat capacity data.

On the other hand, the long-term thermostability of Pels is a crucial aspect from the industrial perspective [16]. To investigate the effect of temperature on enzyme stability, PelN was incubated at different temperatures at pH of 9.8, and the samples were drawn at various time points. The results indicated that the recombinant PelN was considerably stable at 45 and 50°C, with activities decreased to only 76.1 and 46.5%, respectively, after 12-h incubation. The half-life values of PelN were approximately 2,500 min at 45°C, 550 min at 50°C, 50 min at 55°C, and 16.5 min at 60°C, respectively (Figure 6b). The effect of temperature on protein structural stability was studied by carrying out the DSC experiment, and the melting temperature midpoint (T_m_) of the purified PelN was found to be 70.5°C (Figure 7). Besides, thermally induced denaturation was irreversible for PelN, as no detectable energy transition was observed in a second scan of the corresponding samples.

#### Substrate specificity

The enzyme activities on pectins were also investigated under the standard condition. As shown in Table 3, PelN exhibited relatively high activity on methylated substrates. On pectin with relatively low degree (20–34%) of methylation, the remaining specific activity of PelN was approximately 100% of that on PGA. High methylated pectin (55–70%) resulted in slight inhibition of the PelN activity, which was still higher than 74%.

**Table 3:** Effects of different chemicals on the activity of the purified recombinant PelN*

**Substrate**		Relative activity **	***K***_***m***_	***V***_***max***_	***k***_**cat**_	***k***_**cat**_**/*K***_***m***_
		**(%)**	**(g L**^**−1**^**)**	**(mol mg**^**−1**^ **min**^**−1**^**)**	**(s**^**−1**^**)**	**(L s**^**−1**^ **g**^**−1**^**)**
PGA		100.0 ± 4.2	3.48 ± 0.35	4.12 ± 0.77	3.29 × 10^6^	9.45 × 10^5^
Pectin	20–34%	100.9 ± 6.1	15.06 ± 1.24	15.16 ± 0.31	1.21 × 10^7^	8.03 × 10^5^
	55–70%	74.3 ± 2.6	3.32 ± 0.07	1.15 ± 0.09	0.92 × 10^6^	2.77 × 10^5^

*Data represent means ± SD (n = 3) relative to the reference samples;

**The specific activity of PelN on PGA under the standard conditions was used as the reference (100%).

Subsequently, the dependence of the enzyme reaction rate on substrate concentration was investigated under the standard conditions using PGA and both the types of pectins, respectively. The kinetic parameters of PelN were found to vary considerably on different substrates. The corresponding *V*_*max*_ of PelN on PGA was 4.12 mol mg^−1^ min^−1^, which was 3.7-fold higher than that on 20–34% methylated pectin, and 3.6-fold lower than that on highly methylated pectin. The affinity of PelN, indicated by *K*_*m*_, for 20–34% methylated pectin, was lower than that for the other two substrates, whereas the catalytic efficiency, indicated by *k*_cat_/*K*_*m*_, on PGA was similar to that on low methylated pectin and was much higher than that on highly methylated pectin.

### Treatment of ramie bast fiber

**Figure 8:**
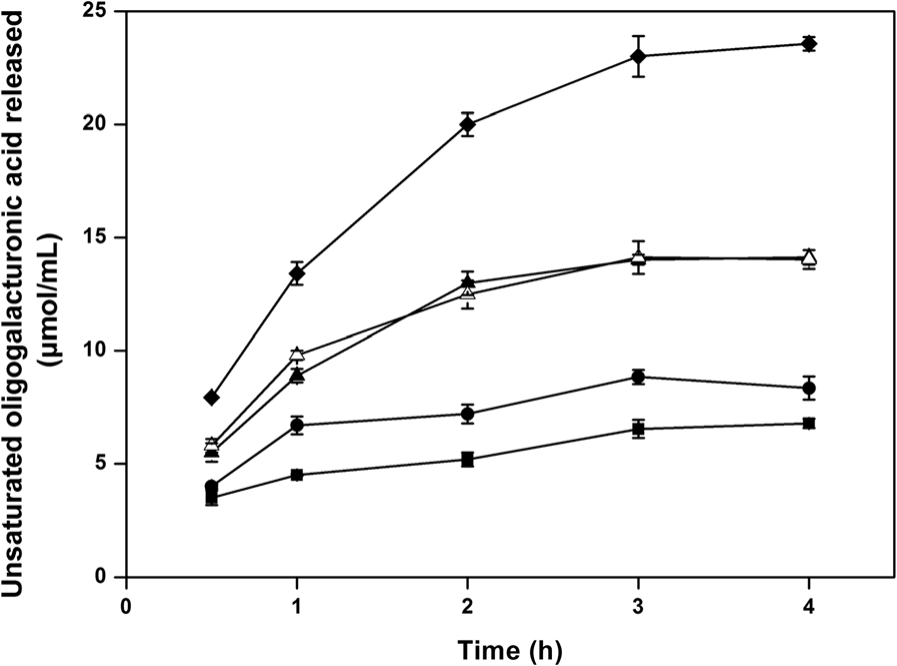
**Unsaturated oligogalacturonic acid released from ramie bast fibers with different treatments.** Ramie bast fibers were treated with 100 U ml^−1^ (■),300 U ml^−1^ (●),500 U ml^−1^ (▲) and 700 U ml^−1^ (△) of PelN, and 500 U ml^−1^ of PelN after pre-treatment of 2% NaOH for 0.5 h(♦), respectively. Error bars represent ± SD (n = 3).

**Figure 9:**
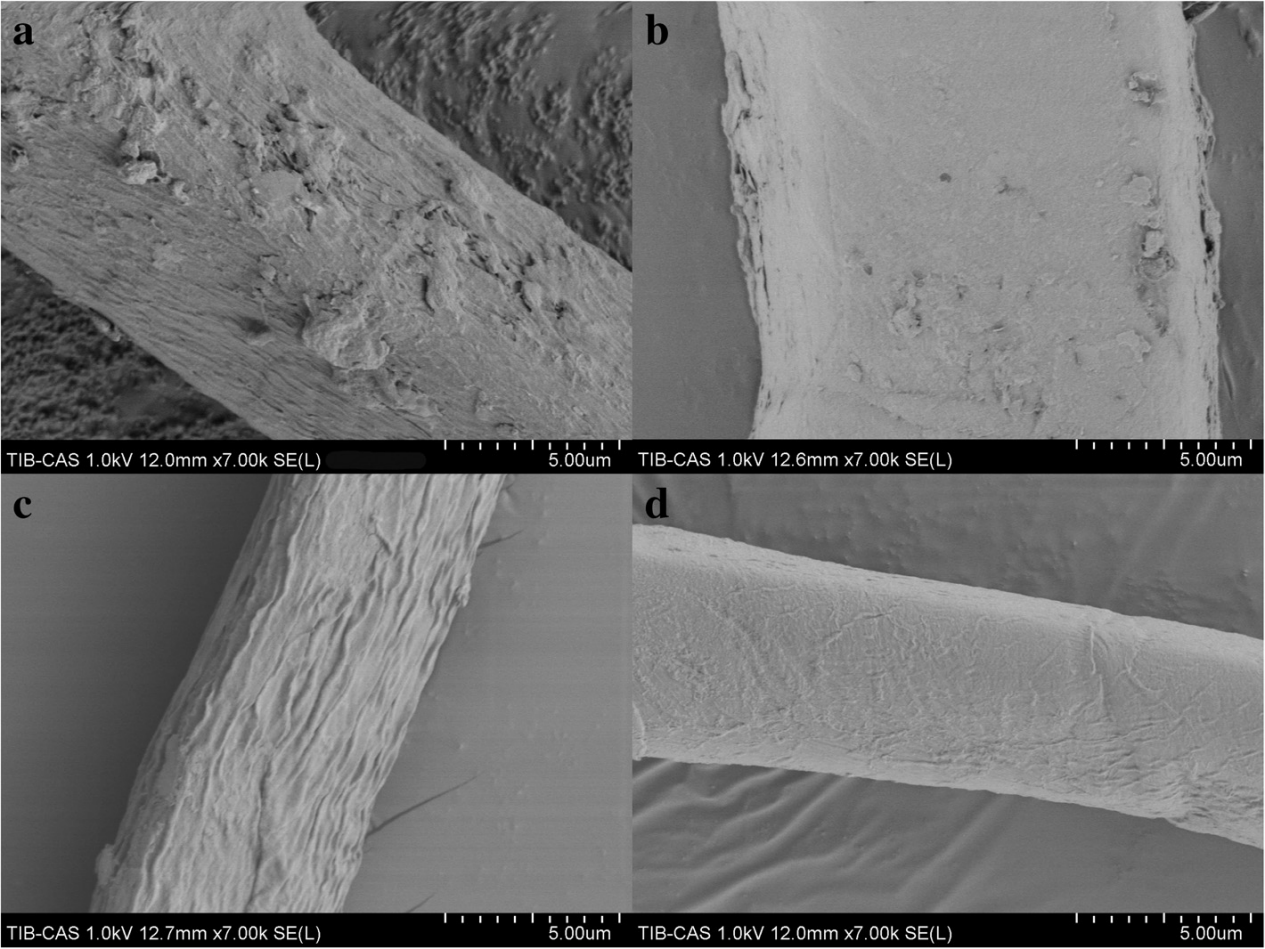
**Scanning electron micrographs of ramie bast fibers. (a)** PL buffer treated fibers (control); **(b)** PelN treated fibers; **(c)** 2% NaOH treated fibers; **(d)** 2% NaOH and subsequently PelN treated fibers.

The enzymatic degumming processing of ramie fiber was optimised in terms of the enzyme dose at 50°C. As shown in Figure 8, the unsaturated oligogalacturonic acid releasing peaked at 14.0 μmol mL^−1^ after 3-h treatment with 500U mL^−1^ of PelN, and an increase in either enzyme dose or treatment time had no significant effect on the degumming reactions. However, when chemically pre-treated ramie fibers were subsequently treated with 500U mL^−1^ of PelN at 50°C, the unsaturated oligogalacturonic acid released reached 23.0 μmol mL^−1^ after 3-h of enzymatic treatment. Accordingly, the percent weight loss of ramie fiber treated by 500U mL^−1^ of PelN and the combined biochemical method reached the maximums (12.0% and 30.5%) after 3-h of enzymatic treatment. Compared with chemical treatment, in which the percent weight loss of ramie fiber peaked at 19.0% after 6-h of 2% NaOH treatment at 90°C, the combined biochemical method seemed to be more effective and economical. The fibers were also observed using an SEM, and the sample after biochemical treatment showed the smoothest surface in our experiments (Figure 9), suggesting that the gum-like material could be mostly removed by the combined method.

## Discussion

In this study, the encoding gene of a Pel from a *Paenibacillus* strain previously isolated from high alkaline soil was cloned. Heterologously production of this protein was accomplished using the recombinant *E. coli* BL21 (DE3). Compared with the highest yield of Pel in *E. coli* previously reported by Fang *et al*. [17], the extracellular Pel production achieved in the present study exhibited an 8.54-fold increase, and the secretory capability increased from 69.3 to 89.2%. Pels have also been expressed in other expression systems, such as *B. subtilis*[18,19] and *Pichia pastoris*[20,21], among which the highest yield reported previously was 2,138 U mL^−1^ in *B. subtilis* after 44-h cultivation in a 7.5-L fermentor [22]. This is 1.15-fold lower than the production reported in this study. Moreover, compared with the *B. subtilis* expression system, the production of Pel using *E. coli* offers several important advantages. These include more reliable carriers of recombinant plasmids and no sporulation under stress conditions, which are propitious for large-scale industrial production. The secretory expression strategy of PelN proposed in this study achieved a relatively higher secretion capability. This strategy has not only overcome the limitations of inclusion body formation, but also significantly simplified the downstream processing of this enzyme, as the fermentation broth could be directly used in fiber degumming.

Furthermore, biochemical properties of the recombinant PelN were also investigated in the present study. The optimal pH of this enzyme showed a similarity with the majority of alkaline Pels previously reported [7]. Nevertheless, except for a few hyperthermophilic Pels [8,23], the optimal temperature (65°C) of PelN was much higher than those of the common Pels from *P. amylolyticus*[12], *Bacillus* sp. [9] and *B. subtilis* 168 [24] (40-50°C). Since a moderate temperature (37–55°C) has often been applied in practical bioscouring processing, most of the enzymatic assays were carried out at 45 or 50°C in the present study, at which the relative activity was only 44.5% or 54.7% of the maximum, but was still practical.

The effect of calcium ions that play an important role in the activity of PelN was also investigated. On one hand, the activity of this enzyme was further elevated with the increase of Ca^2+^ concentration until apparent saturation was reached. On the other hand, the activity of PelN could not be detected in the presence of metal cation chelator. Zheng *et al*. [25] found that in crystals of different Pels of the PL1 family, the numbers of Ca^2+^ ions and the corresponding binding residues differed from each other. This suggests that the variety of the numbers of Ca^2+^-binding residues may be responsible for different modes of the calcium dependence of Pels. In the case of PelN, three aspartates (Asp^151^, Asp^173^, and Asp^177^) were predicted to bind to two Ca^2+^ ions [Additional file 1] by MSA analysis (Figure 1). Moreover, Asp^173^ was found to have a more significant impact on the catalysis than the other two aspartates, presumably due to its potential interaction with both Ca^2+^ ions. Similar Ca^2+^-binding mode, residues and dependence were also observed in Pel from a marine strain *T. maritima*[8]. As aspartates are capable of binding to several types of cations, addition of Mg^2+^ and Zn^2+^ could slightly enhance the enzyme activity. This phenomenon has been previously observed in other Pels, such as PelZ from *E. chrysanthemi*[26] and Pel22 from *B. pumilus* BS22 [27], when using various divalent cations as cofactors.

One of the most attractive properties of PelN is its robust thermo-alkalistability. In fiber bioscouring, alkaline environment and moderate temperature are required for effective degumming and fiber quality improvement. Therefore, enzyme stability under such conditions is a key property for commercially viable bioprocesses. Similar to most Pels, PelN showed maximum activity under alkaline conditions. Moreover, this protein was found to be very stable over a wide pH range and exhibited highest stability in stronger alkaline solution (pH 11.0), thereby meeting the requirements of the textile industrial bioscouring processes. As a thermostable candidate, PelA from the hyperthermophilic bacterium *T. maritima*, with an optimal temperature of 90°C and 2-h half-life at 95°C, has been found to be the most thermoactive Pel characterized to date [8]. Meanwhile, researchers have also made significant efforts to improve the thermostability of Pel. For example, sequence-based knowledge has provided potential amino acid substitutions for stability optimization of PelII from *Xanthomonas campestris*, leading to a 23-fold increase in its half-life (1,292 min) at 45°C [28]. As the half-life of PelN has been demonstrated to be longer than 41 h at 45°C, it can be considered to be an ideal candidate for further engineering for large-scale industrial application when similar strategies are deployed.

Another notable feature of PelN is its activities on both PGA and various methylated pectins. It has been reported that the degree of methylation of naturally occurring pectins is about 70% [7], thus making the robust activity of PelN on highly methylated substrates an attractive property for practical applications. To date, most of the reported Pels have been found to be capable of degrading PGA or low methylated substrates more efficiently than highly methylated pectins (> 22%) [29-32]. Notably, PelN demonstrated a similar activity on PGA and 20–34% methylated pectin, and its activity on 55–70% methylated pectin remained 74%. Comparison of the determined *K*_*m*_ values of the recombinant PelN suggested that the enzyme exhibited an approximately 4.5-fold increase in the affinity for PGA and highly methylated pectin (55–70%) than that for pectin with a low degree (20–34%) of methylation. In contrast, the specificity constant *k*_cat_/*K*_*m*_ values of PelN decreased with the increasing degree of methylation of the substrate. It has been reported that the *K*_*m*_ values of Pels from *Bacillus* sp. [9], *B. licheniformis*[5], *X. campestris*[28] and *Streptomyces* sp. S27 [33] on PGA range from 0.2 and 7.9 g L^−1^. Although the *K*_*m*_ value determined for PelN on PGA falls within this range, it is much higher than those of other bacterial Pels [5,9,28], indicating relatively lower substrate affinity. Interestingly, in the case of PelII from *X. campestris*[28], the *K*_*m*_ value was reported to be only 0.98 g L^−1^, which was 3.6-fold lower than that of PelN, while the *k*_cat_ value of PelII was much lower (10^4^-fold) than that of PelN. Thus, the catalytic efficiency of PelN indicated by *k*_cat_/*K*_*m*_ was more favorable.

In the degumming experiments, the biochemical method developed in our research has proved to be more effective and milder than conventional chemical treatment. Moreover, the total processing time was reduced from 6 h to 3.5 h. In earlier reports, the natural producers of Pels were used directly in the bio-degumming [34-36]. In such cases, inducible Pels and/or other pectin-degrading enzymes are secreted into the media by microorganisms, break down the pectin polymers and then provide nutrients for the producers [1]. The enzyme production and degumming occur in the same process; however, the processing time is significantly prolonged because of bacterial adaptation and the low yield of Pels in low-cost degumming media. For example, it took 28 and 48-h incubation of the alkaliphilic bacteria *Bacillus* sp. NT-39 and NT-76, respectively, to remove the gum of more than 5.0% of the fibers [34], and 24-h incubation of *B. pumilus* to cause the weight loss of the ramie fiber by 25% [36]. Degumming performed by enzymes from fermentation supernatant of recombinant producers was demonstrated to be more practical. Pel168 from the *B. subtilis* 168 was produced by recombinant *P. pastoris*, and used in ramie fiber degumming. The results showed that the textile lost 15% weight after 1-h treatment and only suffered from a minimal detrimental effect for fibers [24]. Admittedly, chemical treatment outperforms enzymatic method in terms of gum removal rate. The combined treatment used in our experiments and other studies [36,37] was demonstrated to considerably reduce the consumption of chemicals and energy, thus making itself more economical and eco-friendly.

## Conclusions

A pectate lyase gene from *Paenibacillus* sp. 0602 was cloned and overproduced in *E. coli* BL21 (DE3). The enzyme exhibited favourable alkalistability, with a maximum residual activity after incubation at pH 11.0 for 1 h, and showed promising thermostability, with the highest activity level at 65°C and long-term half-life at 45 and 50°C. Furthermore, this enzyme was capable of maintaining high specific activities on methylated pectin. In ramie degumming processing, the combined biochemical method with the advantage of reduced process time and energy consumption was found to be a promising substitute to traditional alkaline treatment characterized by high pollution. These findings, together with its high production in the recombinant *E. coli* BL21 (DE3), highlight the industrial application potential of this enzyme as a key enzyme in mild and cost-effective bioresource industries in textile, paper-making, and pectic wastewater treatment.

## Methods

### Strains and culture media

*Paenibacillus* sp. 0602 was isolated from high alkaline soil in Bohai Gulf, and has been deposited in the China General Microbiological Culture Collection Centre (CGMCC No. 5696). *E. coli* BL21 (DE3) was used for recombinant protein expression, and was cultivated in modified Terrific broth (TB) medium (10 g L^−1^ of glycerol, 24 g L^−1^ of yeast extract, 12 g L^−1^ of peptone, 17 mM KH_2_PO_4_, and 72 mM K_2_HPO_4_).

### Nucleic acid manipulation

The chromosomal DNA of *Paenibacillus* sp. 0602 was prepared as previously described [38], which was subsequently used as the template in degenerate PCR and inverse PCR. Degenerate primers *pel*-F and *pel*-R were designed according to the conservative patterns “nviirN” and “v/iWiDH” of Pels with some modifications of those used by Wang *et al.*[39]. To clone the intact ORF of the *pel* gene and its flanking sequences, inverse PCR was performed using the method developed by Ochman *et al*. [40] with primers *pel*-inverse-F and *pel*-inverse-R. The PCR fragment of *pel*N without signal peptide encoding sequence was amplified with PCR using primers *pel*N-F and *pel*N-R, and inserted into the expression plasmid pET-22b(+). The resulting plasmid pET-pelN was transformed into *E. coli* BL21 (DE3). Mutations were introduced using the QuickChange mutagenesis kit, and the mutagenic primers were designed according to the instruction given in the manual. All primers used in this manuscript are listed in [Additional file 2].

### Bioinformatic analysis

Analysis of the product of degenerate PCR was performed using BLAST ( http://www.ncbi.nlm.nih.gov/blast/). The sequence of the product of inverse PCR was respectively analyzed by using the ORF Finder ( http://www.ncbi.nlm.nih.gov/gorf/gorf.html) [41] to identify the complete ORF, the PePPER webserver ( http://pepper.molgenrug.nl/) [42] to predict the upstream elements, and the RibEx program ( http://www.ibt.unam.mx/biocomputo/ribex.html) [43] to identify its transcription terminator. Deduction of amino acid sequence and calculation of molecular mass and isoelectric point of PelN were performed with the DNAMAN 5.0 software (Lynnon Biosoft, Quebec, Canada). The signal peptide was predicted with SignalP ( http://www.cbs.dtu.dk/services/SignalP/) [44] specifically configured for gram-positive bacteria. To identify functional sites of PelN, the MSA of several Pels with solved structures from PL1 family without signal peptides was constructed using the MUSCLE program ( http://www.ebi.ac.uk/Tools/msa/muscle/) [45].

### Protein expression and purification

In shake-flask cultivation, the expression of recombinant PelN using *E. coli* BL21(DE3) was carried out as described previously [39]. The protein in the supernatant was concentrated by ammonium sulphate precipitation, resuspended in buffer A (20 mM sodium phosphate, pH 7.1), and dialyzed against the same buffer. After centrifugation at 15,000 × g for 1 h to remove cell debris and insoluble proteins, the supernatant was loaded to an UNOsphere S column (Bio-Rad) previously equilibrated against buffer A, and eluted using a linear sodium chloride gradient from 0 to 500 mM. The active fractions were pooled and dialyzed against stock buffer (50 mM Hepes-KOH supplemented with 7 mM β-mercaptoethanol and 1 mM EDTA, pH 7.1).

SDS-PAGE analysis was performed using 10% separating gel. After electrophoresis, the protein bands were visualized by Coomassie brilliant blue R-250 staining.

### Enzyme assays

The purified proteins were used in enzyme assays. In the standard condition, the assay mixtures containing 2 mL of 0.2% (w/v) PGA in PL buffer (50 mM glycine–NaOH buffer, pH 9.8) supplemented with 0.5 mM CaCl_2_ were incubated for 15 min at 45°C, and 3 mL of 50 mM phosphoric acid was added immediately to terminate enzymatic reactions. The absorption at 235 nm, indicating the formation of unsaturated products, was spectrophotometrically measured [21]. One unit of enzymatic activity was defined as the amount of enzyme that produced 1 μmol of unsaturated product per minute under the above-mentioned assay conditions.

To characterize the biochemical properties of PelN, the influence of pH, temperature, various chemicals, and substrates with different degrees of methylation was comprehensively investigated. The optimal pH of the purified recombinant PelN was determined at 45°C and a pH range of 7.1–12.0 using 50 mM Tris–HCl buffer (pH 7.1–9.0), 50 mM glycine–NaOH buffer (pH 9.0–11.0), and 50 mM Na_2_HPO_4_–NaOH buffer (pH 11.0–12.0), respectively. The optimal temperature was determined in PL buffer at different temperatures in the range of 35–70°C. To analyze the effects of different metal ions and other chemicals on the activity of PelN, 0.5 and 1 mM Mg^2+^, Mn^2+^, Cu^2+^, Zn^2+^, Fe^2+^, K^+^, Ba^2+^, and Ca^2+^, as well as EDTA, Triton X-100, and Tween-20 were added to the PL buffer, respectively, and 0.2% PGA (w/v) was used as the substrate. To investigate the influence of the substrate methylation on the activity of PelN, PGA was replaced with two types of pectins (Sigma) with low degree (20–34%) and high degree (55–70%) of methylation, respectively. To determine the kinetic parameters, the enzymes were incubated at 45°C with PGA or pectins at concentrations ranging from 0.05 to 1 g L^−1^.

### Stability measurement

For the analysis of pH-dependent stability, the enzyme was incubated at 45°C for 1 h at a pH range of 7.0–12.0 using 50 mM sodium citrate–citric acid buffer (pH 4.0–6.6), 50 mM Tris–HCl buffer (pH 6.6–9.2), 50 mM glycine–NaOH buffer (pH 9.2–11.0), and 50 mM Na_2_HPO_4_–NaOH buffer (pH 11.0–12.0), respectively. The residual activity of each sample was then measured under the standard assay condition. To determine the thermostability of PelN, the half-life of inactivation was measured using the following procedure: PelN was pre-incubated in PL buffer at 45, 50, 55, and 60°C, respectively. The samples were drawn at different time points and subjected to standard enzyme assays.

To perform the differential scanning calorimetry (DSC) experiments, the purified PelN enzymes were diluted to 0.2 g L^−1^ using 10 mM Na_2_HPO_4_–NaOH buffer (pH 9.8). Calorimetric measurements were carried out as described by Solbak *et al*. [46] with necessary modification. The DSC experiments were performed on a Nano DSC apparatus (TA Instruments, USA) with a scan rate of 36°C h^−1^ at 1 atm. The amount of heat absorbed or released during protein thermal denaturation was measured. The raw data were converted to an apparent molar heat capacity by correcting for the buffer–buffer scanning curve and dividing each data point by scan rate and the amount of proteins in the sample cell.

### Degumming and scanning electron microscopy of ramie bast fibers

Degumming was performed according to Kapoor *et al*. [37] with certain modification. Decorticated ramie fibers were boiled in water for 15 min, and then dried before treatments. Degumming of ramie bast fibers was carried out in 250-ml Erlenmeyer flasks containing 2 g of dried raw fibers. In chemical treatment, fibers were incubated in 100 mL of 2% NaOH solution at 90°C. To balance the activity and stability of PelN, the enzyme-involved treatments were carried out at 50°C. In enzyme treatment, fibers were incubated in 100 mL of PL buffer (with 0.5 mM CaCl_2_) containing different doses of PelN. Controls were treated under the same condition without the addition of PelN. In the combined biochemical treatment, fibers were treated with 2% NaOH solution at 90°C for 0.5 h, washed with water twice and subsequently incubated with the optimised PelN dose (500 U mL^−1^) at 50°C. In enzyme-involved treatments, the increase of absorbance at 235 nm, indicating the releasing unsaturated oligogalacturonic acid from fibers, was measured to monitor the pectolytic processing.

Scanning electron microscopy (SEM) was performed to observe the variation of surface structures of differently treated fibers. The samples were prepared as described earlier [37], and then were observed under SEM (Hitachi SU8010, Japan).

### Nucleotide sequence accession number

The DNA sequence of the *pel*N gene from *Paenibacillus* sp. 0602 has been deposited in the GenBank database under the accession number.

## Competing interests

The authors declare financial competing interests. The contents of this manuscript have been partially patented by Tianjin Institute of Industrial Biotechnology, Chinese Academy of Sciences (Application Number 201310155628.4).

## Authors’ contributions

XL carried out the cloning, the purification of PelN, DSC experiment, degumming processing and SEM as well as drafted the manuscript. HW investigated the heterologous expression and the biochemical characterization of PelN protein. CZ offered valuable experimental protocols of the degumming processing. JL, YM and JS critically revised and corrected the manuscript. All authors have read and approved the final manuscript.

## Supplementary Material

Additional file 1 Relative activities of the PelN mutants. Click here for file

Additional file 2 Forward (−F) and reverse (−R) primers used in PCRs and site-directed mutagenesis. Click here for file
