# Supplementary material for: Cloning, expression and characterization of a pectate lyase from Paenibacillus sp. 0602 in recombinant Escherichia coli
Source: BMC Biotechnol. 2014 Mar 10;14:18. doi: 10.1186/1472-6750-14-18 (PMC4007691; doi:10.1186/1472-6750-14-18)
Supplement: Additional file 1 — Relative activities of the PelN mutants. [file 1472-6750-14-18-S1.docx]

**Additional file 1**

| Mutants | Predicted function of mutated residues | Relative activities |
| --- | --- | --- |
| Native Psp62PelN |  | 100% |
| R275A | Catalytic base | <0.01% |
| R280A | Neutralizes substrate charges | 1.5% |
| K244A | Catalytic acid | 1.4% |
| D151A | Binds Ca^2+^ 1 | 1.8% |
| D173A | Binds Ca^2+^ 1 and 2 | <0.01% |
| D177A | Binds Ca^2+^ 1 | 0.5% |
