# Supplementary material for: Cloning, expression and characterization of a pectate lyase from Paenibacillus sp. 0602 in recombinant Escherichia coli
Source: BMC Biotechnol. 2014 Mar 10;14:18. doi: 10.1186/1472-6750-14-18 (PMC4007691; doi:10.1186/1472-6750-14-18)
Supplement: Additional file 2 — Forward (−F) and reverse (−R) primers used in PCRs and site-directed mutagenesis. [file 1472-6750-14-18-S2.docx]

**Additional file 2**

| Primers | Nucleotide sequence* |
| --- | --- |
| *pel*-F | 5＇-AA(C/T)GT(A/T/C/G)AT(A/T/C)AT(A/T/C) (A/C)G(A/T/C/G) AA-3＇ |
| *pel*-R | 5＇-TG(A/G)TC(G/A/T)ATCCA(A/T/C/G)AC/T-3＇ |
| *pel*-inverse-F | 5＇-ATAAAGCCTATGACGGACTTGTGGATTCG-3＇ |
| *pel*-inverse-R | 5＇-AGGTGAGGTCTGCGCCGCACTTGGAATCT-3＇ |
| *pel*N-F | 5＇-AGCACCATGGCGGGCAATGCAGATTACAAT-3＇ |
| *pel*N-R | 5＇-CGAACGCTCGAGTTAATAGCTCGTCTTCAGCCAGTT-3＇ |
| D173A-F | 5＇-CCTTCAATAAAGCCTAT**GCA**GGACTTGTGGATTCG-3＇ |
| D173A-R | 5＇-CGAATCCACAAGTCC**TGC**ATAGGCTTTATTGAAGG -3＇ |
| D151A-F | 5＇-CGACAAGAACGATTGG**GCA**TACATTACACTAGAGGAAAGC-3＇ |
| D151A-R | 5＇-GCTTTCCTCTAGTGTAATGTA**TGC**CCAATCGTTCTTGTCG-3＇ |
| R280A-F | 5＇-CCGGATGCCGCGACTG**GCA**GGTGGTAATGCAC-3＇ |
| R280A-R | 5＇-GTGCATTACCACC**TGC**CAGTCGCGGCATCCGG-3＇ |
| K244A-F | 5＇-CTGGTTCGCAGAAA**GCA**GGTCATCTTGTCGG-3＇ |
| K244A-R | 5＇-CCGACAAGATGACC**TGC**TTTCTGCGAACCAG-3＇ |
| D177A-F | 5＇-CCTATGACGGACTTGTG**GCA**TCGAAGAAGGGAACC-3＇ |
| D177A-R | 5＇-GCAGTCGCGGCAT**TGC**GTCCTGAATGTCTTTATAG-3＇ |
| R275A-F | 5＇-CTATAAAGACATTCAGGAC**GCA**ATGCCGCGACTGC-3＇ |
| R275A-R | 5＇-GCAGTCGCGGCAT**TGC**GTCCTGAATGTCTTTATAG-3＇ |

^*^Mutated nucleotides are written in bold characters.
